# Increased NUSAP1 expression is associated with lymph node metastasis and survival prognosis in bladder urothelial carcinoma

**DOI:** 10.1038/s41598-022-11137-4

**Published:** 2022-04-29

**Authors:** Jian Hou, Zhenquan Lu, Xianhua Liu, Bingfeng Luo, Genyi Qu, Yong Xu, Cheng Tang

**Affiliations:** 1 Department of Urology, Zhuzhou Central Hospital, Zhuzhou, 412007 China; 2 Division of Urology, Department of Surgery, The University of Hongkong-Shenzhen Hospital, Shenzhen, 518000 China; 3 Department of Pathology, Maternity and Child Care Centers in Fujian Province, Fuzhou, 350000 China

**Keywords:** Tumour biomarkers, Urological cancer

## Abstract

The main route of metastasis of bladder urothelial carcinoma is through lymph nodes; however, its exact mechanism remains unclear. In this study, we found an association of nucleolar and spindle associated protein 1 (NUSAP1) expression with BUC tissues along with lymph node metastasis and the survival prognosis. A total of 178 pathological specimens following radical bladder cancer resection were obtained. NUSAP1 expression was analyzed by immunohistochemistry. We evaluated the correlation between clinicopathological characteristics and NUSAP1 expression. Logistic regression was used to determine the independent variables that influenced lymph node metastasis. Uni- and multi-factorial Cox regression methods were used to determine the prognostic value of NUSAP1 expression in urothelial carcinoma of the bladder. High expression of NUSAP1 in BUC was not significantly related to the patient’s gender, age, or tumor number (*p* > 0.05), however was significantly associated with pathological grade, tumor diameter, pathological stage, and lymph node metastasis (*p* < 0.05). Lymph node metastasis was significantly correlated with pathological stage, pathological grade, tumor number, tumor diameter, and NUSAP1 expression (*p* < 0.05); only NUSAP1 expression was an independent predictor of lymph node metastasis in BUC (OR:1.786, 95% CI 1.229–2.596, *p* = 0.002). In addition, high NUSAP1 expression was an independent prognostic predictor for BUC. In BUC, NUSAP1 showed high expression and was significantly associated with lymph node metastasis, pathological stage, pathological grade, and tumor diameter. NUSAP1 was an independent predictor of lymph node metastasis and prognosis in BUC; higher expression indicated poorer prognosis of BUC patients.

## Introduction

Currently, worldwide, bladder cancer is the most prevalent disease^1^. Among these bladder malignancies, BUC (bladder urothelial carcinoma) accounts for more than 90% of the total. The main route of metastasis is through the lymph nodes, which manifests first as pelvic lymph node metastasis. Risk factors for bladder cancer include smoking, urinary tract infections, and occupational exposure hazards^2^. Patients who receive severe bladder cancer surgery in addition to pelvic lymph node dissection still have a positive risk of lymph node metastasis ranging from 13 to 40%^3^. The absence or presence of lymph node metastasis has an extremely important effect on the treatment strategy and survival prognosis. Although some progress has been made in recent years in surgical procedures, radiotherapy, chemotherapy, and immunotherapy, the 5-year survival and prognosis of bladder cancer show little improvement. Therefore, the search for new tumor markers for the early diagnosis and clinical prognostic assessment of bladder urothelial carcinoma is critical.

Mitosis is an integral cell function that requires great accuracy throughout the entire process to ensure correct and stable chromosome replication^4^. NUSAP1 is a recently discovered microtubule-binding protein with a molecular weight of 55 kD. It is selectively expressed during cell proliferation and is an important regulatory molecule that ensures a proper cell cycle may be included in mitosis^5^. Increased expression of NUSAP1 is closely associated with tumor development and has been reported in prostate cancer, breast cancer, oral squamous cell carcinoma, and cervical cancer^6–9^. However, its role in bladder urothelial carcinoma and its clinical evaluation remain unknown. Therefore, we examined the NUSAP1 expression and its value for prognostic assessment in urothelial carcinoma of the bladder.

## Methods

### Specimens and data collection

Pathological specimens were collected after radical cystectomy and standard lymph node dissection for bladder urothelial carcinoma between January 2015 and December 2016 at Zhuzhou Central Hospital. All patients received standard treatment. Inclusion criteria: all patients included in this study were diagnosed with bladder urothelial carcinoma based on histopathology and had no distant metastasis. Exclusion criteria: patients receiving preoperative adjuvant chemotherapy, radiotherapy, immunotherapy or targeted therapy. And a total of 25 patients were excluded. In addition, 47 BUC-positive lymph nodes and their corresponding adjacent normal bladder tissue samples were obtained as control specimens. A total of 178 specimens (132 males, 46 females) were collected, and the corresponding clinical data of the patients including the number of tumors, size, grade, stage, gender, age, and pelvic lymph node metastasis were recorded (Table 1). and all patients were informed consent for study participation. The present study design was approved by the ethics committee of Zhuzhou Central Hospital, all methods were performed in accordance with the relevant guidelines and regulations, and the research were been performed in accordance with the Declaration of Helsinki.

**Table 1:** Clinicopathological parameters of 178 patients with urothelial carcinoma of the bladder.

Parameter	Number
**Age (y)**	
Mean ± SD	62.37 ± 10.04
Range	34–79
**Gender**	
Male	132 (74.2)
Female	46 (25.8)
**Tumor size (cm)**	
Mean ± SD	3.41 ± 1.30
Range	1.1–6.1
**Multiplicity**	
Single	66 (37.1)
Multiple	112 (62.9)
**Tumor grade [n (%)]**	
Low	27 (15.2)
High	151 (84.8)
**Pathological stage (pT) [*n* (%)]**	
T1-T2	61 (34.3)
T3-T4	117 (65.7)
**Lymph node status**	
Negative	131 (73.6)
Positive	47 (26.4)

### Production of tissue microarray

The paraffin-embedded specimens were collected from the pathology department and resliced for H&E staining. To validate the representative areas for building the tissue core, senior pathologists inspected the H&E-stained sections, and the samples in wax blocks were marked according to the sections. A self-made tissue core construction apparatus was used to punch holes of 2 mm diameter in a new empty white wax block in which 5 × 8 tissue cores could be placed in each recipient block. After the tissue cores were accurately placed into the small holes of the perforated recipient wax block according to the pre-designed sequence, they were sequentially operated on until all of them were planted in the perforated recipient wax block. Finally, the wax block was re-fused. A paraffin slicer was used for continuous sectioning of the tissue core wax blocks to obtain slices with 4 µm thickness.

### Immunohistochemistry

Sections were dewaxed in xylene and hydrated in gradient alcohol, subjected to antigen repair at high temperature and pressure for two minutes, followed by washing thrice with PBS for three minutes each. Next, sections were incubated with 3% peroxidase blocking solution at room temperature for 10 min, followed by washing thrice with PBS for 3 min each. Subsequently, these sections were incubated with normal goat serum at room temperature for 10 min, followed by incubation with NUSAP1 primary antibody (Abcam, cat No: ab137230, 1:200 dilution) overnight at 4 °C. Next day, the sections were washed thrice with PBS for three minutes each and incubated with DAKO ChemMate EnVision reagent (secondary antibody, DAKO, USA) (added dropwise) at room temperature for 30 min. The sections were washed thrice with PBS for three minutes each. The color was developed by dropwise addition of freshly prepared DAB solution. We then rinsed the sections with tap water and restained them with hematoxylin; 1% hydrochloric acid alcohol was used for fractionation for (a few seconds). Finally, gradient alcohol was used to dehydrate and dry, xylene to clear and neutral gum to seal the sections. The samples were observed under a microscope.

### Determination of results

Positive expression of NUSAP1 was defined as the presence of yellowish or brownish granules in the nucleus of the cells. Five representative high magnification fields, with 100 cells per field, were imaged for each section; positive cells and staining intensity percentages were separately scored. The following criteria were used for scoring: (1) staining degree: 0 for no staining, 1 for weak staining (yellowish or yellowish to brownish staining), 2 for moderate staining (between the two), and 3 for strong staining (yellowish to brownish staining); (2) the percentage of stained cells: 0 for 5%, 1 for 5–25%, 2 for 25–50%, 3 for 50–75%, and 4 for > 75%. By the summation of scores for the staining degree and percentage of stained cells, the total score (ranging from 0 to 7) for the sample was calculated. A score of 0 was considered as negative (−), 1 to 2 as weakly positive (+), 3 to 5 as positive (++), and 6 to 7 as strongly positive (++++)^10^. The above results were independently assessed by two pathologists under double-blinded conditions. The fluorescence intensity was determined using ImageJ v.1.8.0 software^11^.

### Statistical analysis

Graphpad prism 9.0 (GraphPad software company, San Diego, California, USA) and packages of the SPSS 25.0 software were used for statistical analyses. The significance of the difference was evaluated by parametric one-way analysis of variance (ANOVA) and multiple comparison tests for the comparison of measurement data. Independent samples chi-square test was used for the comparison of qualitative data. Logistic regression analysis was used for evaluating the predictors of lymph node metastasis. Kaplan–Meier survival curve was used to describe the relationship between the 5-year recurrence-free survival and overall survival in BUC. The univariate Cox regression analysis included gender, pathological grade, age, tumor diameter, number of tumors, lymph node metastasis, pathological stage, and NUSAP1 expression for prognosis of BUC patients; statistically significant parameters from univariate analyses were included for further multifactorial Cox regression analyses. *p* < 0.05 was considered as statistically significant. And further analyze the relationship between NUSAP1 expression and the overall survival rate of bladder cancer through the PrognoScan online database.

### Ethics approval and consent to participate

The present study design was approved by the ethics committee of Zhuzhou Central Hospital, and the research were been performed in accordance with the Declaration of Helsinki and all patients were informed consent for study participation.

## Results

### Patient information

The results of the clinical indices, including age, gender, tumor diameter, tumor number, pathological grade of the tumor, pathological stage of the tumor, and lymph node metastasis for 178 patients in this study are provided in Table 1.

### Differences in NUSAP1 expression between bladder urothelial carcinoma, positive lymph nodes, and normal bladder tissues

**Figure 1:**
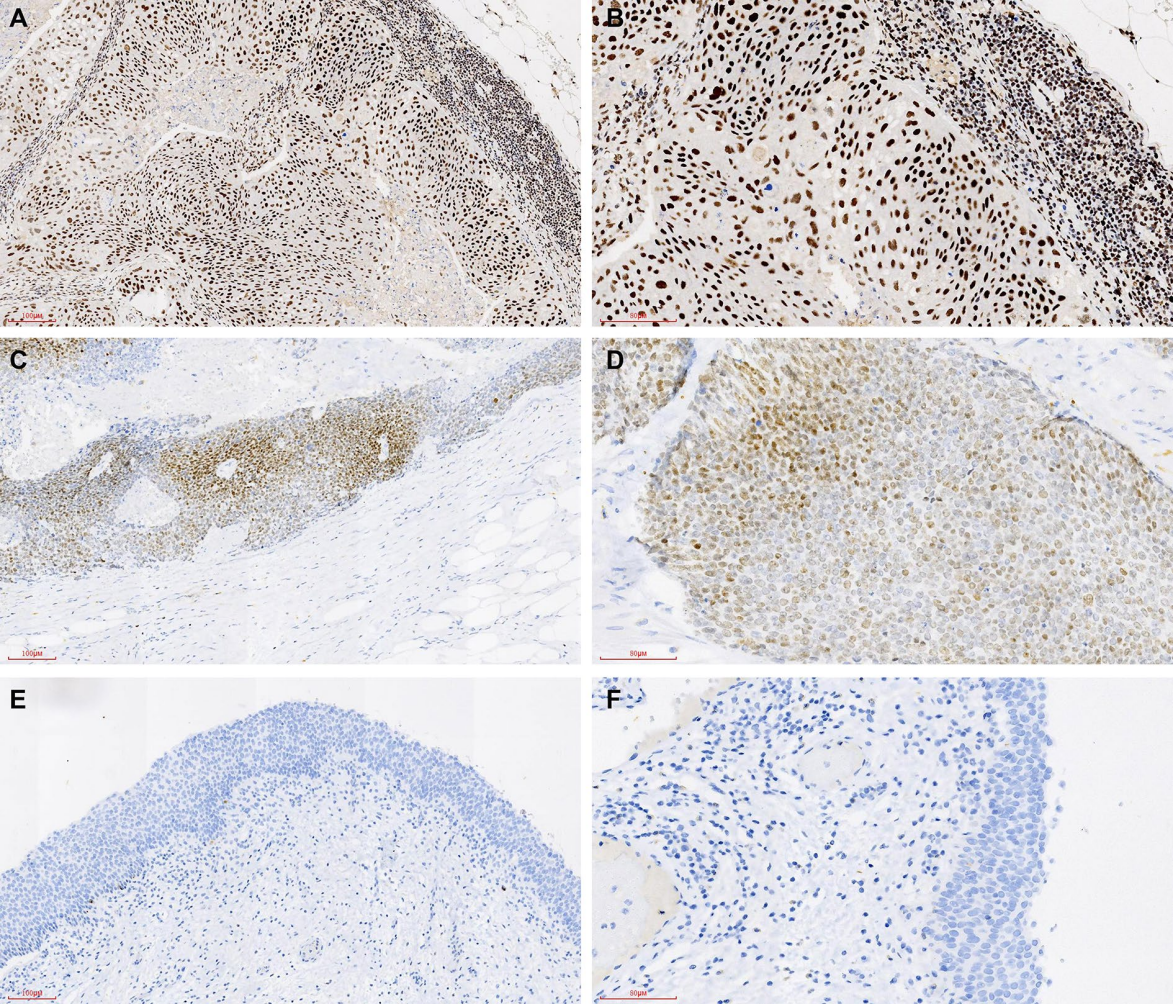
NUSAP1 expression in bladder cancer positive lymph node tissues was significantly higher as compared to the corresponding BUC primary foci tissue; NUSAP1 expression in BUC tissue was significantly higher as compared to the corresponding normal bladder tissues “(**A**,**B** Positive lymph nodes; **C**,**D** BUC; **E**,**F** Normal bladder tissues; **A**,**C**,**E**, ×100; **B**,**D**,**F**, ×200)”.

**Figure 2:**
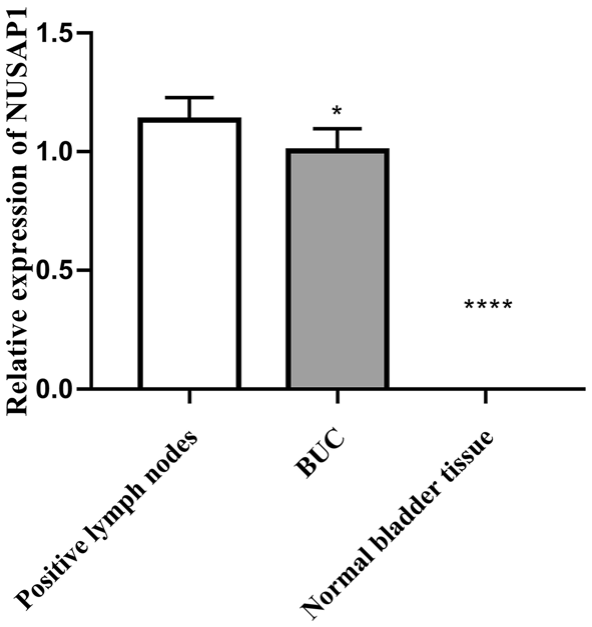
Relative fluorescence density of NUSAP1 in BUC. Scale bar = 50 um. Scale bar = 10 um. The data were analyzed by one-way ANOVA (*P > 0.05, BUC compared with the Positive lymph nodes ***P < 0.001 Normal bladder tissue compared with the BUC and Positive lymph nodes group).

Primarily, NUSAP1 was localized to the cytoplasm of the positive cells. Immunohistochemistry was performed for 47 pathological lymph node samples and the corresponding adjacent normal bladder tissue samples and compared with the correspondingly matched BUC primary foci tissues. 91.49%, 17.02%, and 82.98% of positive lymph nodes, normal bladder tissues, and BUC tissues, respectively, were positive for NUSAP1 expression; the difference between these two was statistically significant (*p* < 0.05; see Table 2, Figs. 1, 2).

**Table 2:** NUSAP1 expression in BUC and normal bladder tissues.

Group	NUSAP1 expression				Positive rate (%)
	−	+	++	+++	
Normal bladder tissue	39	6	2	0	17.02
BUC	8	14	17	8	82.98
Positive lymph nodes	4	8	15	20	91.49

BUC vs. normal bladder tissue, *P* < 0.05; Positive lymph nodes vs. BUC, *P* < 0.05; Positive lymph nodes vs. normal bladder tissue, *p* < 0.05.

### Relationship between NUSAP1 expression and pathological parameters of patients with BUC

**Figure 3:**
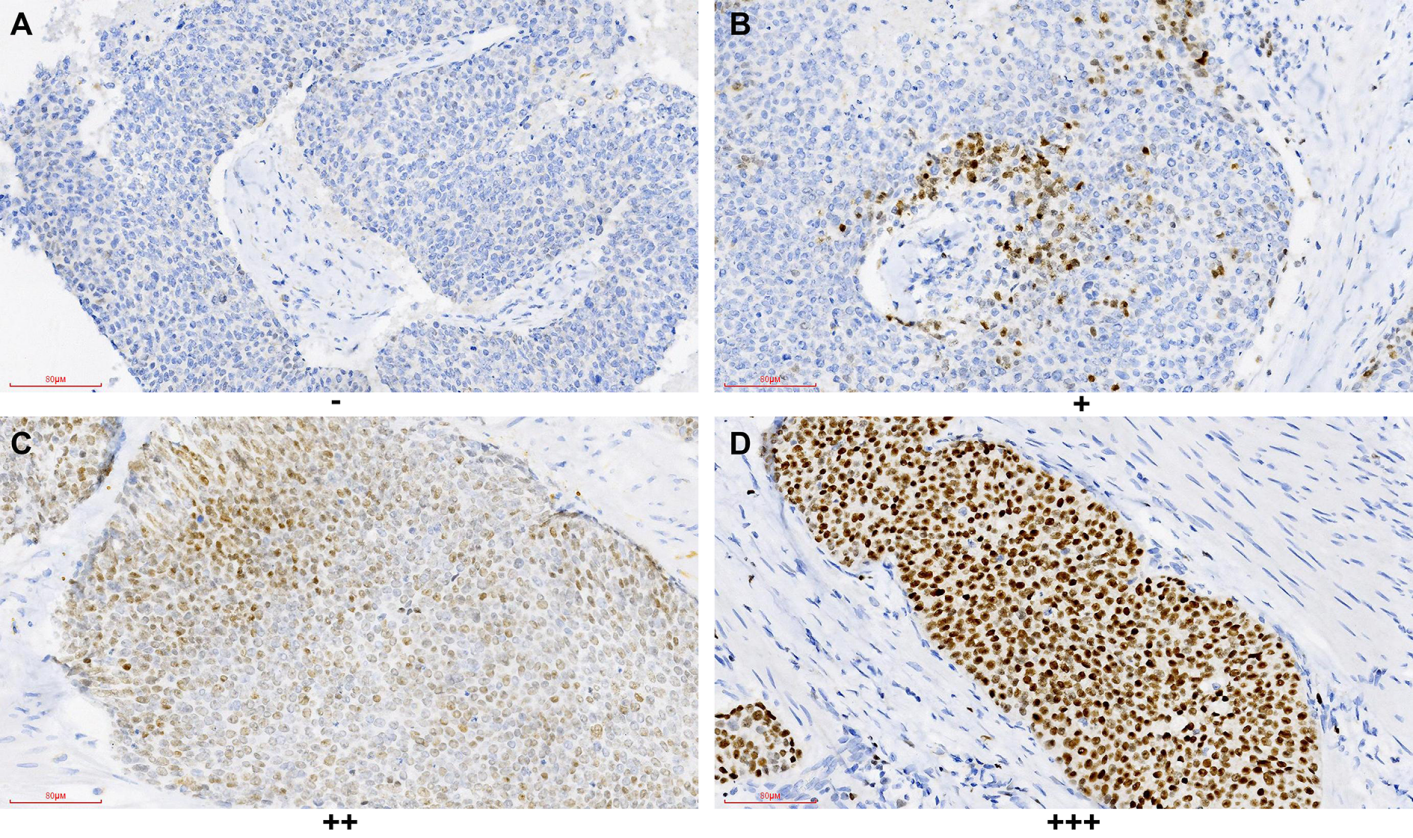
NUSAP1 expression in BUC tissues; strong staining for NUSAP1 was mostly observed in tissues with high-grade, high staged, and lymph node metastasis-positive specimens (×200).

**Figure 4:**
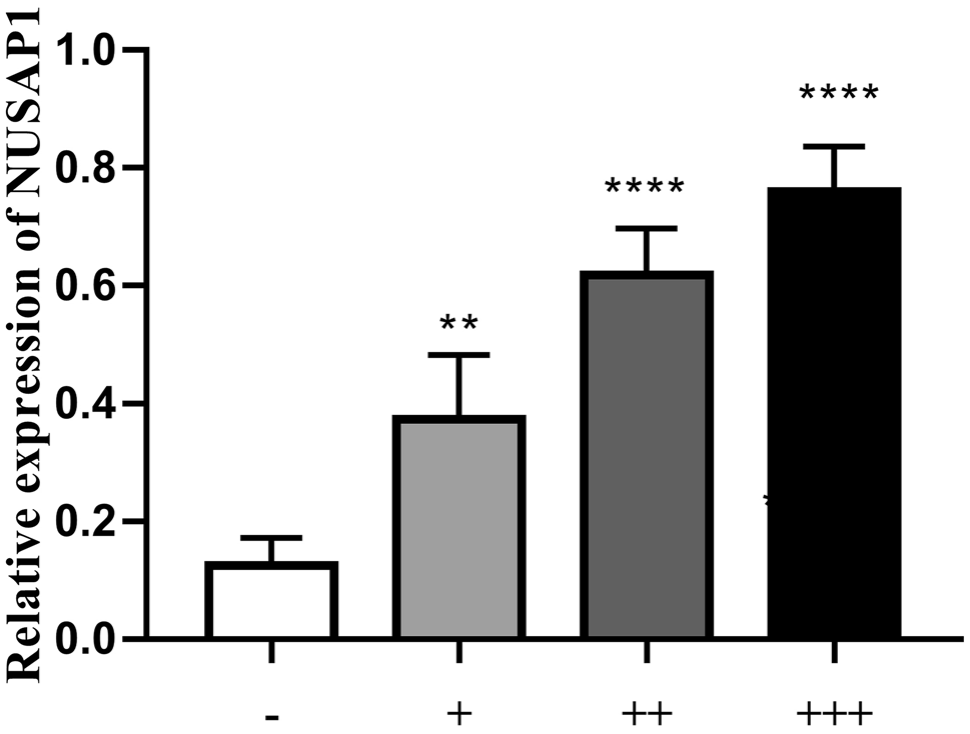
The photographs of the NUSAP1-immunoreactive cells in BUC. Scale bar = 10 um. The data were analyzed by one-way ANOVA (***P < 0.001 compared with the control group; **P < 0.01 compared with the control group).

NUSAP1 expression was significantly correlated with lymph node metastasis, pathological stage, pathological grade, and tumor diameter, (*p* < 0.05, see Table 3), while there was no significant association with patient gender, age, or tumor number (*p* > 0.05, see Table 3.) Strong NUSAP1 staining was observed in specimens with large tumor diameter, high stage, high grade, and positive lymph node metastasis (see Figs. 3, 4).

**Table 3:** Association of NUSAP1 expression with the clinicopathological features of BUC patients.

Variables	N	NUSAP1 expression				*p* value
		−	+	++	+++	
Total, n (%)	178	40 (22.5)	51 (28.7)	44 (24.7)	43 (24.1)	
**Age (yrs)**						0.209
< 60	73 (41.0)	20 (50.0)	17 (33.3)	15 (34.1)	21 (48.8)	
≥ 60	105 (59.0)	20 (50.0)	34 (66.7)	29 (65.9)	22 (51.2)	
**Gender**						0.488
Female	46 (25.8)	9 (22.5)	12 (23.5)	10 (22.7)	15 (34.9)	
Male	132 (74.2)	31 (77.5)	39 (76.5)	34 (77.3)	28 (65.1)	
**Tumor size (cm)**						0.017
< 3	74 (41.6)	23 (57.5)	22 (43.1)	19 (43.2)	10 (23.3)	
≥ 3	104 (58.4)	17 (42.5)	29 (56.9)	25 (56.8)	33 (76.7)	
**Multiplicity**						0.066
Single	66 (37.1)	22 (55.0)	17 (33.3)	14 (31.8)	13 (30.2)	
Multiple	112 (62.9)	18 (45.0)	34 (66.7)	30 (68.2)	30 (69.8)	
**Tumor grade [n (%)]**						0.001
Low	27 (15.2)	14 (35.0)	6 (11.8)	4 (9.1)	3 (7.0)	
High	151 (67.5)	26 (65.0)	45 (88.2)	40 (90.9)	40 (93.0)	
**Pathological stage (pT) [*n* (%)]**						0.027
T1-T2	61 (34.3)	20 (50.0)	17 (33.3)	16 (36.4)	8 (18.6)	
T3-T4	117 (65.7)	20 (50.0)	34 (66.7)	28 (63.6)	35 (81.4)	
**Lymph node status**						< 0.001
Negative	131 (73.6)	36 (90.0)	43 (84.3)	29 (65.9)	23 (53.5)	
Positive	47 (26.4)	4 (10.0)	8 (15.7)	15 (34.1)	20 (46.5)

### Effect of NUSAP1 expression and other clinicopathological parameters on pelvic lymph node metastasis in BUC

Subsequent subgroup analysis based on the absence or presence of lymph node metastasis showed that in BUC, the pelvic lymph node metastasis was significantly associated with pathological stage, pathological grade, tumor number, tumor diameter, and NUSAP1 expression (*p* < 0.05, see Table 4), but not with the gender and age of the patients (*p* > 0.05, see Table 4). The level of NUSAP1 expression was an independent risk factor for lymph node metastasis in BUC (*p* < 0.05, see Table 5).

**Table 4:** Analysis of factors influencing lymph node metastasis in urothelial carcinoma of the bladder.

Variables	N	Lymph node metastasis		*p* value
		Negative	Positive	
Total, n (%)	178	131 (73.6)	47 (26.4)	
**Age (yrs.)**				
< 60	73 (41.0)	52 (39.7)	21 (44.7)	0.551
≥ 60	105 (59.0)	79 (60.3)	26 (55.3)	
**Gender**				
Female	46 (25.8)	28 (21.4)	18 (38.3)	0.053
Male	132 (74.2)	103 (78.6)	29 (61.7)	
**Tumor size (cm)**				
< 3	74 (41.6)	64 (48.9)	10 (21.3)	0.001
≥ 3	104 (58.4)	67 (51.1)	37 (78.7)	
**Multiplicity**				
Single	66 (37.1)	56 (42.7)	10 (21.3)	0.009
Multiple	112 (62.9)	75 (57.3)	37 (78.7)	
**Tumor grade [n (%)]**				
Low	27 (15.2)	26 (19.8)	1 (2.1)	0.004
High	151 (84.8)	105 (80.2)	46 (97.9)	
**Pathological stage (pT) [*n* (%)]**				
T1-T2	61 (34.3)	54 (41.2)	7 (14.9)	0.001
T3-T4	117 (65.7)	77 (58.8)	40 (85.1)	
**NUSAP1 expression**				
−	40 (22.5)	36 (27.5)	4 (8.5)	< 0.001
+	51 (28.7)	43 (32.8)	8 (17.0)	
++	44 (24.7)	29 (22.1)	15 (31.9)	
+++	43 (24.2)	23 (17.6)	20 (42.6)

**Table 5:** Logistic regression analysis of factors influencing lymph node metastasis in urothelial carcinoma of the bladder.

Variables	OR (95% CI)	*P* value
Tumor size (< 3 vs. ≥ 3)	2.053 (0.871–4.839)	0.100
Multiplicity (single vs. multiple)	1.973 (0.839–4.640)	0.120
Tumor grade (low vs. high)	4.822 (0.572–40.625)	0.148
pT stage (T1-T2 vs. T3-T4)	2.277 (0.866–5.983)	0.095
NUSAP1 (−/+/++/+++)	1.786 (1.229–2.596)	0.002

### Analysis of factors influencing recurrence-free and overall 5-year survival rate after BUC surgery

**Figure 5:**
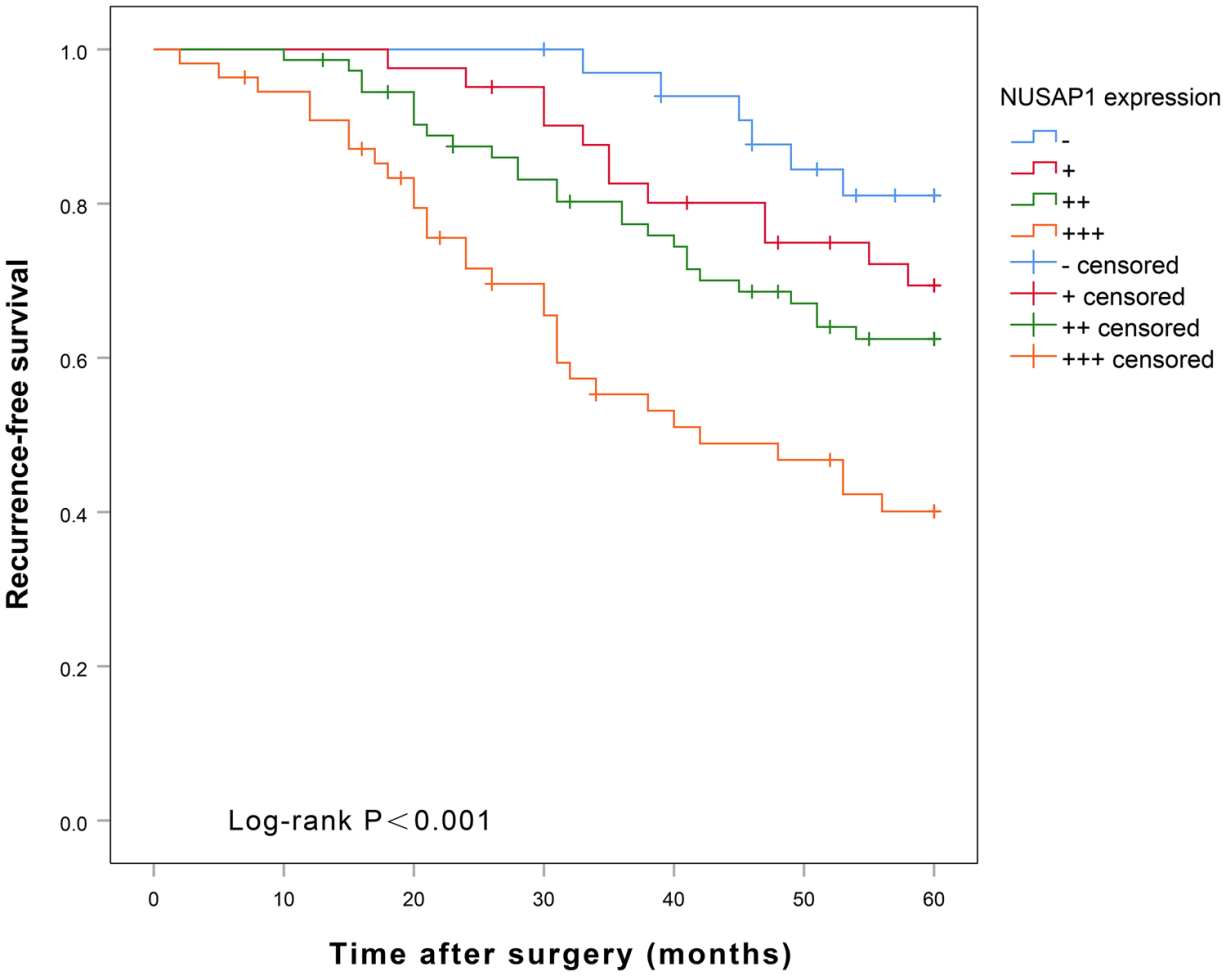
NUSAP1 expression levels and 5-year recurrence-free survival curves for BUC patients after radical surgery.

**Figure 6:**
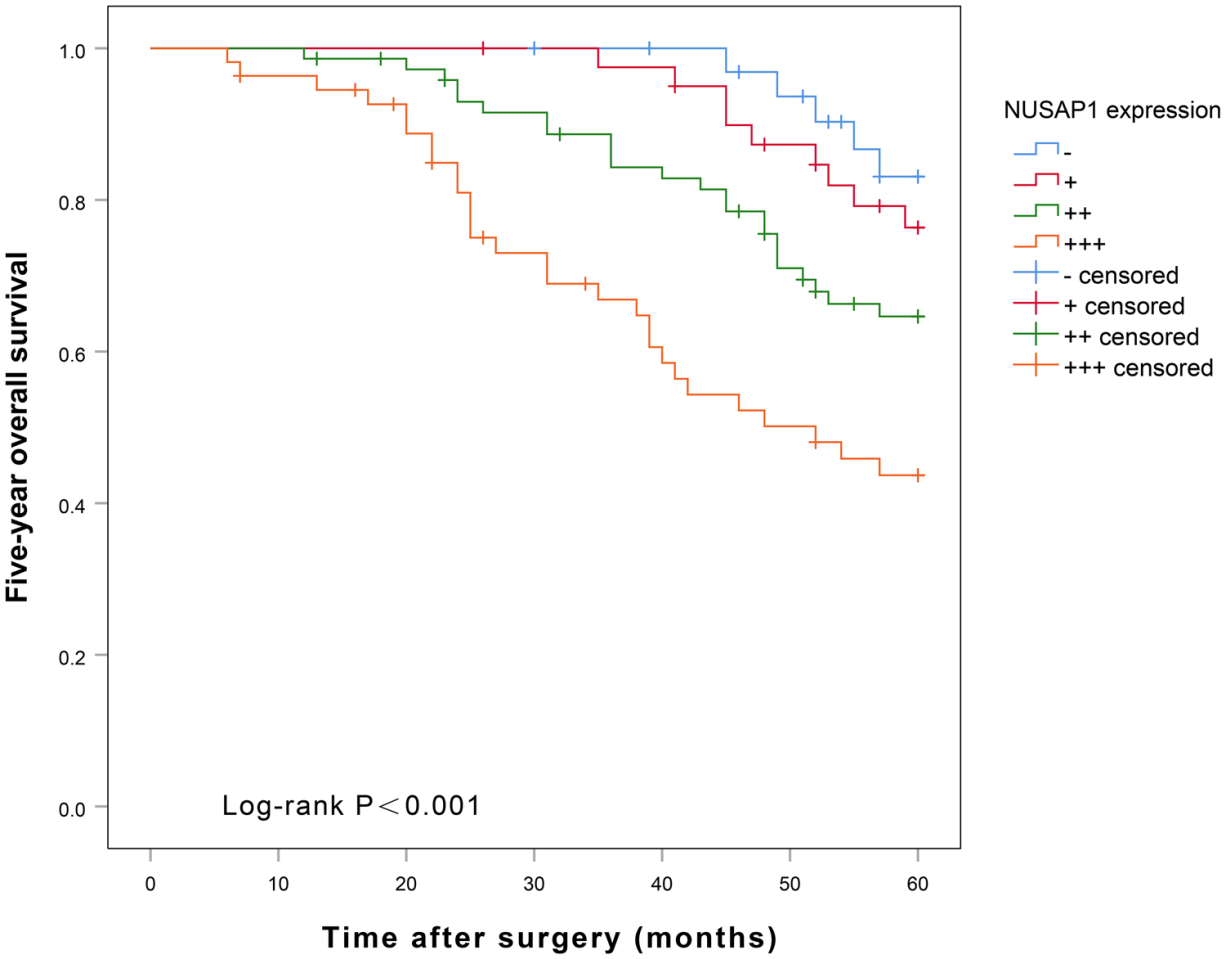
NUSAP1 expression levels and 5-year overall survival curves for BUC patients after radical surgery.

**Figure 7:**
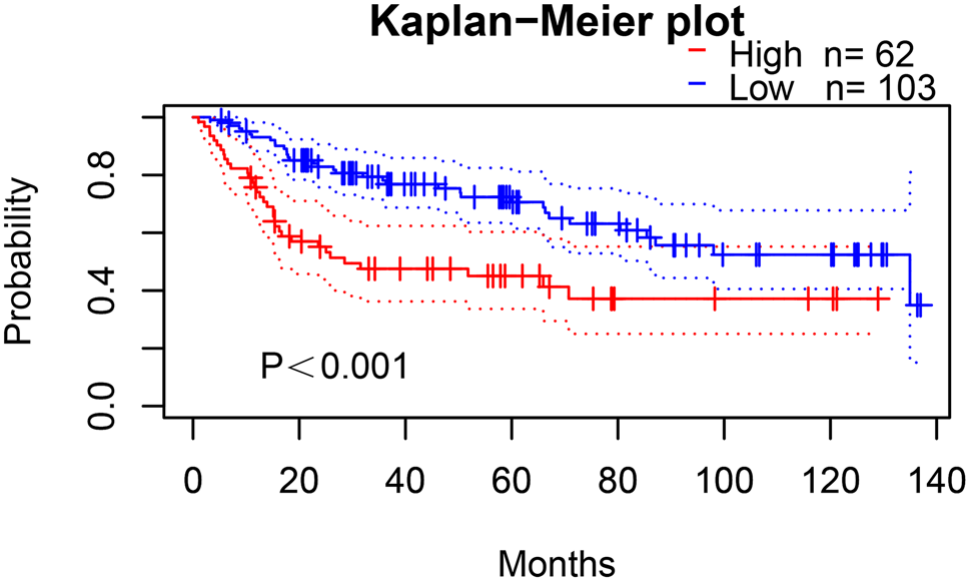
Survival analysis of patients with BLCA obtained from PrognoScan (GSE13507).

Univariate Cox regression analysis showed that pathological grade, tumor diameter, tumor number, lymph node metastasis, pathological stage, and NUSAP1 expression were significantly associated with the 5-year recurrence-free survival (RFS) (*p* < 0.05, see Table 6) and 5-year overall survival (OS) (*p* < 0.05, see Table 7) after radical surgery for BUC. The multifactorial Cox regression analysis showed that only high NUSAP1 expression could predict shorter RFS at 5 years (*p* < 0.05, see Table 6), and both high pathological stage and high NUSAP1 expression predicted shorter 5 year-OS (*p* < 0.05, see Table 7) after BUC surgery. Kaplan–Meier plot of 5-year RFS after radical surgery for BUC (Fig. 5) showed that patients with high NUSAP1 expression had a significantly shorter recurrence-free survival time as compared to those with low expression of NUSAP1 (*p* = 0.001). The OS curves (Fig. 6) showed that patients with high NUSAP1 expression had a significantly shorter survival time relative to those with low NUSAP1 expression = 0.004). PrognoScan online analysis showed that the overall survival rate of bladder cancer patients with high NUSAP1 expression was significantly shorter than that of patients with low NUSAP1 expression ((*p* < 0.001) (Fig. 7).

**Table 6:** Univariate Cox and multifactorial analysis for factors influencing 5-year-recurrence-free survival after surgery for BUC.

Variables	Univariate analysis		Multivariate analysis	
	HR (95% CI)	*P* value	HR (95% CI)	*P* value
Age (yrs.; < 60 vs. ≥ 60)	1.551 (0.924–2.604)	0.097		
Gender (male vs. female)	0.708 (0.427–1.176)	0.182		
Tumor size (< 3 vs. ≥ 3)	2.124 (1.235–3.651)	0.006	1.297 (0.741–2.269)	0.363
Multiplicity (single vs. multiple)	1.921 (1.126–3.277)	0.017	1.426 (0.819–2.484)	0.209
Tumor grade (low vs. high)	5.497 (1.724–17.525)	0.004	3.066 (0.937–10.032)	0.064
pT stage (T1-T2 vs. T3-T4)	2.913 (1.557–5.451)	0.001	1.860 (0.977–3.539)	0.059
Lymph node status (negative vs. positive)	2.562 (1.571–4.180)	< 0.001	1.296 (0.763–2.201)	0.338
NUSAP1 (−/+/++/+++)	1.732 (1.381–2.170)	< 0.001	1.559 (1.219–1.995)	< 0.001

**Table 7:** Univariate Cox and multifactorial analysis for factors influencing 5-year-overall survival after surgery for BUC.

Variables	Univariate analysis		Multivariate analysis	
	HR (95% CI)	*P* value	HR (95% CI)	*P* value
Age (yrs.; < 60 vs. ≥ 60)	1.509 (0.863–2.638)	0.149		
Gender (male vs. female)	0.683 (0.398–1.170)	0.165		
Tumor size (< 3 vs. ≥ 3)	2.388 (1.306–4.366)	0.005	1.403 (0.753–2.613)	0.287
Multiplicity (single vs. multiple)	1.960 (1.099–3.495)	0.023	1.366 (0.747–2.495)	0.311
Tumor grade (low vs. high)	6.948 (1.693–28.511)	0.007	3.828 (0.910–16.105)	0.067
pT stage (T1-T2 vs. T3-T4)	3.363 (1.649–6.859)	0.001	2.105 (1.016–4.363)	0.045
Lymph node status (negative vs. positive)	2.796 (1.658–4.714)	< 0.001	1.372 (0.776–2.425)	0.277
NUSAP1 (−/+/++/+++)	1.737 (1.361–2.217)	< 0.001	1.539 (1.176–2.014)	0.002

## Discussion

Urothelial carcinoma of the bladder accounts for approximately 95% of bladder cancers and is the leading cause of urinary system-related deaths^12^. Although radical surgery, neoadjuvant radiotherapy, immunotherapy, and other novel therapies are widely used in the treatment of bladder cancer, prognosis remains typically poor with a high recurrence rate after the surgery; patients with BUC have a recurrence rate as high as 70% within 5 years after surgery^13^. Lymph node metastasis is the most common metastatic route which affects the postoperative treatment, the survival time of bladder cancer patients, and increases the likelihood of recurrence after surgery^14^. Therefore, accurate detection of lymph node metastasis and early treatment before surgery is crucial to reduce recurrence and improve the overall prognosis of bladder cancer patients. Studies have reported that the circulating tumor cell (CTC) is a noninvasive prognostic marker and can be used as a prognostic marker for the risk stratification of NMIBC patients^15^. At the same time, studies have found that CTC can identify ultra-high-risk NMIBC patients who need to be closely monitored for local recurrence and/or disease progression, and guide patients for closer follow-up, early radical surgery, and even systemic treatment^16^. In addition, studies have reported that the baseline basophil count can predict the recurrence of bladder cancer patients undergoing BCG infusion chemotherapy^17^. In addition, studies have reported that the Vesical Imaging Reporting and Data System (VI-RADS) score is adopted to provide bladder cancer staging, which can accurately distinguish between NMIBC and MIBC, and is helpful to guide patients in the choice of preoperative surgery^18–20^.

Molecular tumor markers for prognostic evaluation, repetition, and progression of BUC have a significant impact^21–23^. NUSAP1 is a microtubule-related protein that plays a key role in chromosomal segregation, spindle assembly, cytoplasmic division, and cross-linking of microtubules^24^. Depletion of NUSAP1 leads to disassembly of the mitotic spindle, abnormal chromosome segregation, and defective cytoplasmic division. Conversely, overexpression of NUSAP1 leads to microtubule bundling and cell cycle arrest at the G2/M checkpoint^4^. Thus, NUSAP1 has a significant role in the mitotic process of proliferating cells. In this study, by immunohistochemical staining, we found that NUSAP1 expression was significantly high in BUC samples as compared to the normal bladder epithelial tissues, and the high expression of NUSAP1 was significantly associated with the tumor diameter, lymph node metastasis, pathological grade, and the pathological stage.

FAM101B is a TGF-β1 signaling effector^25^ and a downstream potential regulator of NUSAP1 in tumor progression. TGF-β1 is a major regulator of epithelial-to-mesenchymal transition (EMT), a process that enables tumor invasion and metastasis^26^. In BUC, NUSAP1 regulates EMT through the TGF-β1 signaling pathway, and inhibition of TGF-β1 receptor (TGFBR1) can significantly inhibit the invasion and metastasis in BLCA cells^27^.

Unfortunately, the failure of several preoperative examinations in detecting micro-metastases is the main reason for the early recurrence of BUC after surgery. The occurrence of micro-metastases is closely related to the clinicopathological characteristics of the tumor (including pathological stage and pathological grade); the more advanced the pathological stage and higher the pathological grade, the more likely is the micro-metastases and recurrence after surgery^28^. In this study, we found that high NUSAP1 expression was significantly correlated with tumor pathological stage, lymph node metastasis, and pathological grade; NUSAP1 expression was significantly higher in patients with T3-T4 TNM stage, higher pathological grades, and positive lymph nodes (*p* < 0.05). Therefore, tumors with high NUSAP1 expression have a higher probability for the formation of micro-metastases and recurrence after surgery. The present study also showed that NUSAP1 was an independent predictor of lymph node metastasis in BUC. NUSAP1 can enhance the aggressiveness of tumor cells and participate in the astrocytoma progression by activating the Hedgehog signaling pathway^29^. In addition, O-GlcNAcylation in bladder cancer can promote NUSAP1 expression, and in turn enhance the proliferation and inhibit apoptosis in bladder cancer HT-1376 and T24 cells. NUSAP1 is involved in spindle assembly and its overexpression leads to microtubule aggregation into bundles, which in turn causes M-phase block in several cells^4^. This may be one of the reasons for the late pathological stage and the high degree of pathological grade, which in turn predisposes to the formation of micro-metastases in the patients.

High expression of NUSAP1 can be used as a prognostic biomarker for glioblastoma multiforme, pancreatic cancer, and colon cancer^30–32^. NUSAP1 high expression was an independent predictor for 5-year RFS risk in BUC and the 5-year OS was independent of the higher pathological stage and high NUSAP1 expression. In addition, patients with high expression of NUSAP1 had significantly lower RFS and OS than those with low NUSAP1 expression. Therefore, high NUSAP1 expression could be used as an independent prognostic indicator in BUC patients.

The weaknesses of this study include the following. First, this study lacks cellular and molecular experiments for further treatment. And second, it was a retrospective study, so it had an inherent bias. Finally, the comparison of the tissues was all from patients with BUC rather than normal patients. Comparison with normal bladder tissue from patients without BUC would be more precise. However, at present, studies have reported that circulating tumor cells (CTC) can be used as a non-invasive prognostic marker for bladder cancer. This study is based on histological biopsy as an invasive examination.

In conclusion, NUSAP1 levels were elevated in BUC and were significantly associated with tumor diameter, pathological grade, pathological stage, and lymph node metastasis. In addition, NUSAP1 was an independent predictor for lymph node metastasis and BUC. NUSAP1 expression levels may enable clinicians to evaluate the prognosis of BUC patients and may provide a new avenue for the development of future treatment strategies.

## Data Availability

All data generated or analyzed during this study are included in this article.
